# Opposite functions of GSN and OAS2 on colorectal cancer metastasis, mediating perineural and lymphovascular invasion, respectively

**DOI:** 10.1371/journal.pone.0202856

**Published:** 2018-08-27

**Authors:** Jin Cheon Kim, Ye Jin Ha, Ka Hee Tak, Seon Ae Roh, Yi Hong Kwon, Chan Wook Kim, Yong Sik Yoon, Jong Lyul Lee, Yangsoon Park, Seon-Kyu Kim, Seon-Young Kim, Dong-Hyung Cho, Yong Sung Kim

**Affiliations:** 1 Department of Surgery, University of Ulsan College of Medicine, Seoul, South Korea; 2 Institute of Innovative Cancer Research, Asan Medical Center, Seoul, South Korea; 3 Department of Pathology, University of Ulsan College of Medicine, Seoul, South Korea; 4 Medical Genomics Research Center, Korea Research Institute of Bioscience & Biotechnology, Daejeon, South Korea; 5 School of Life Science, Kyungpook National University, Daegu, Korea; University of Kansas School of Medicine, UNITED STATES

## Abstract

The present study aimed to identify molecules associated with lymphovascular invasion (LVI) and perineural invasion (PNI) and to examine their biological behavior in colorectal cancer (CRC). LVI- and PNI-associated molecules were identified and verified using sequential processes including (1) identification of 117 recurrence-associated genes differentially expressed on RNA-seq analysis using primary cancer tissues from 130 CRC patients with and without systemic recurrence; (2) analysis of molecules associated with LVI and PNI; (3) assessment of biological properties by measuring proliferation, anoikis, invasion/migration, epithelial-mesenchymal transition and autophagy flux; and (4) verification of disease-free survival using public datasets. Gelsolin (GSN) and 2'-5'-oligoadenylate synthetase 2 (OAS2) were associated with PNI and LVI, respectively. Invasion potential was >2-fold greater in GSN-overexpressing LoVo cells than in control cells (*p*<0.001–0.005), whereas OAS2-overexpressing RKO cells showed reduced invasion (*p*<0.001–0.005). GSN downregulated E-cadherin, β-catenin, claudin-1 and snail, and upregulated N-cadherin and ZEB1, whereas OAS2 overexpression had the opposite effects. Several autophagy-related proteins including ATG5-12, ATG6/BECN1, ATG7 and ATG101 were downregulated in GSN-overexpressing LoVo cells, whereas the opposite pattern was observed in OAS2-overexpressing RKO cells. Patients with low GSN expression had significantly higher 5-year recurrence-free survival (RFS) rates than those with GSN overexpression (73.6% vs. 64.7%, *p* = 0.038), whereas RFS was longer in patients with OAS2 overexpression than in those with underexpression (73.4% vs. 63.7%, *p* = 0.01). In conclusion, GSN and OAS2 were positively and negatively associated with recurrence, respectively, suggesting their potential value as predictors of recurrence or therapeutic targets in CRC patients.

## Introduction

Approximately 25% of patients with colorectal cancer (CRC) have metastatic disease at the time of diagnosis, and 30–50% of CRC patients undergoing curative resection develop metastasis or recurrence during the follow-up period [1]. Three principal channels of metastasis, namely, hematogenous, lymphatic and peritoneal routes of dissemination, have been established. Metastatic routes of spread are frequently associated with histological traces of lymphovascular invasion (LVI), perineural invasion (PNI), extramural vascular invasion and tumor budding. Among these parameters, LVI and PNI are recognised as category I prognostic factors representing aggressive CRC (AJCC cancer staging, 8th ed, https://cancerstaging.org/).

LVI is defined as the invasion of tumor cells into thin-walled small vessels including capillaries, post-capillary venules and lymphatics. A systematic analysis including 9881 CRC patients and 19 relevant studies showed that LVI is significantly associated with poor survival outcomes [2]. The incidence of LVI reported in that study ranged from 5.2% to 30.3%. Several molecular markers were introduced to help identify key genes and pathways driving LVI. Among these markers, CDKN2A hypermethylation was identified in a meta-analysis as significantly associated with LVI in addition to lymph node metastasis and proximal tumor location [3].

PNI is another clear route of metastatic spread, although the role of nerves in cancer progression remains relatively unknown [4]. PNI includes tumor cells within the three layers of the peripheral nerve sheath or in close proximity to a nerve and involving at least 1/3 of its circumference. The incidence of PNI is 18.2% overall in CRC cohorts, and it is more frequent in rectal cancer than in colon cancer; it is an independent prognostic factor for survival in multivariate analysis [5]. The infiltration of the tumor microenvironment by nerves suggests that tumor neoneurogenesis is an active process facilitating cancer progression [6]. Synuclein-γ overexpression is observed in 61% of patients with pancreatic cancer and correlates with major invasive parameters, including PNI and lymph node metastasis [7].

The molecular mechanism underlying the association between metastasis and the main routes of cancer progression remains unclear [4]. The primary aim of this study was to identify molecules associated with LVI and PNI as major channels of CRC metastasis and to examine their biological behavior. In addition, the prognostic significance of these molecules for the prediction of recurrence in CRC patients with LVI and PNI on histological analysis and their value for the early detection of CRC were examined.

## Materials and methods

### Patient enrolment, sample acquisition, and main scheme

**Fig 1:**
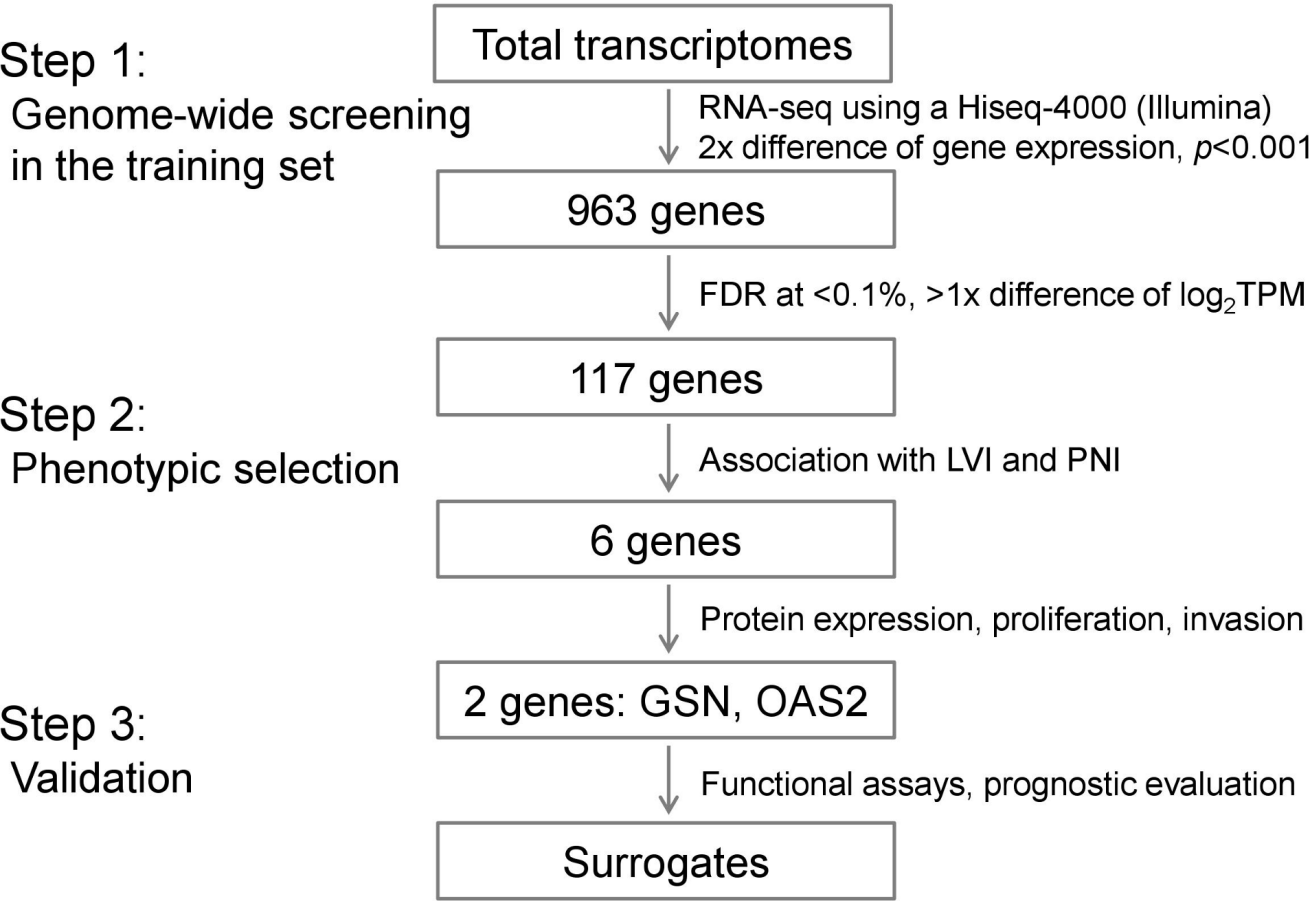
Summary of the algorithm used for the three-step process to identify surrogate genes of CRC recurrence via LVI and PNI with sequential outcome. TPM, transcripts per kilobase million.

Primary tumor samples were obtained from 130 patients with colorectal adenocarcinomas and used for RNA extraction. The clinicopathological features of the patients are summarized in S1 Table. All samples were collected at Asan Medical Center (Seoul, Korea) after obtaining written consent from patients and stored in a -210°C liquid nitrogen tank. Patients with hereditary CRC (familial adenomatous polyposis and hereditary non-polyposis CRC) and those with cancers arising from inflammatory bowel disease were excluded. The patients were divided into two groups as follows: patients without systemic recurrence for more than 5 years (n = 72) and patients with systemic recurrence (n = 58). Systemic recurrence was defined as synchronous or metachronous metastasis excluding locoregional relapse. A summary of the procedure used to identify surrogate genes is shown in Fig 1. The study protocol was approved by the Institutional Review Board of Asan Medical Center (registration numbers: 2018–0087).

### Transcriptome profiling

The sequencing library was prepared using the TruSeq RNA sample preparation kit v2 (Illumina, CA, USA) according to the manufacturer’s instructions after total RNA isolation. The mRNA was purified from total RNA using poly-T oligo-attached magnetic beads, fragmented and converted into cDNA. Sequencing was performed in paired-end reads (2 × 150 bp) using a Hiseq-4000 (Illumina). The reference genome index was built using SAMtools (ver. 0.1.18), and RNA-seq samples were quantified using Kallisto (ver. 0.43.0). To estimate the significance of differences in gene expression between sample subgroups, an EdgeR package with a negative binomial model was applied to detect differentially expressed genes from the count data [8]. Genes were selected using a generalized linear model (GLM) likelihood ratio test that specifies probability distributions according to the mean-variance relationship. Expression differences in genes were considered statistically significant if the *p*-value was <0.001 and the fold difference in expression between two sample groups was ≥2. A total of 963 genes were differentially expressed between patients with and without systemic recurrence (S2 Table). These genes were further narrowed down using a strict false discovery rate (FDR) as <0.1% and differential values of log_2_ transcripts per million (TPM) as >1-fold differences, leaving 117 genes. Then, the log_2_ TPM values of the 117 genes were compared between the two groups according to LVI and PNI.

### Real-time reverse transcription-PCR, transfection, and cloning

The procedures for RNA extraction and quantitative real-time reverse transcription-PCR (RT-PCR) were described previously using the respective primers (S3 Table) [9]. Ten CRC cell lines (ATCC, Manassas, VA, USA) and two control epithelial cell lines (kindly provided by Yonsei University, Seoul, Korea) used were free of mycoplasma and authenticated using purified DNAs on a 3130x1 Genetic analyzer and GeneMapper software 5 (Cosmo Genetech, Seoul, Republic of Korea). They were cultured in RPMI-1640 supplemented with 10% (v/v) fetal bovine serum and 1% (w/v) penicillin and streptomycin following the provider’s recommendations. CRC cell lines with minimal mRNA expression for specific molecules were selected for gene transfection or knockdown (S1 Fig). The cDNAs of six genes [gelsolin (GSN), 2'-5'-oligoadenylate synthetase 2 (OAS2), UDP glucuronosyltransferase family 1 member A6 (UGT1A6), palmdelphin (PALMD), synuclein gamma (SNCG) and heat shock protein family B member 6 (HSPB6); cDNA: OriGene, Rockville, MD, USA] were amplified by PCR and subcloned into DDK-tagged pCMV6-entry for stable transfection (OriGene). Cells transfected with pCMV6-entry vector were used as the control group. Transient transfection was performed in cancer cells using Lipofectamine 2000 (Invitrogen, Carlsbad, CA, USA), and stably expressing cells were generated by G418 selection for 10 days, with at least two clones selected for each cell line. For target gene knockdown, the corresponding human-specific siRNAs (Dharmacon/Seoulin Bioscience, Hwaseong-si, Korea; Invitrogen) were transfected into cells using RNAiMax transfection reagent (Invitrogen). Cells transfected with negative control siRNA (siNC: BIONEER, Daejeon, Korea) was used as controls. The protein expression of the six genes was confirmed by western blot analysis (S2 Fig).

### Immunoassays

Protein extracts from tumor tissues and cultured cells (approximately 50 μg) were quantified by using Bradford solution (BioRad, Hercules, CA, USA). The samples were resolved by SDS/10% polyacrylamide gel electrophoresis, and transferred to polyvinylidene difluoride (Millipore, Billerica, MA, USA) membrane for western blot analysis. Membranes were consecutively incubated with primary antibodies. The specific complexes were detected using SuperSignal west pico kit (Thermo Scientific, Rockford, IL, USA). Deparaffinized tissues were subjected to immunohistochemistry (IHC, concentrations: 1:40000 for GSN and 1:50 for OAS2) based on the labeled streptavidin–biotin method using a DAKO LSAB^®^ kit (DAKO, Carpinteria, CA, USA). The immunoreactivity was classified into three categories according to intensity (0, negative; 1, weak; 2, moderate; 3, strong) and proportion (0, ≤5%; 1, 6–30%; 2, 31–60%; 3, >60%). For immunoprecipitation and indirect immunofluorescence, cells were washed three times with ice-cold PBS and lysed with RIPA buffer and phosphatase inhibitor single-use cocktail (ThermoFisher Scientific, Rockford, IL, USA). The cell lysate was centrifuged at 15,000 g for 15 min at 4°C and supernatant was incubated with anti-GSN mouse monoclonal antibody (Abnova, Taipei, Taiwan). Normal mouse IgG (Santa Cruz, Dallas, Texas) was used as a negative control. After overnight incubation at 4°C with gentle rotation, a protein A/G plus agarose was added (Santa Cruz) and incubated at 4°C for 4 h with gentle rotation. The remained beads were resuspended with 1× SDS sample buffer and boiled for 5 min. The supernatant was further analyzed by western blotting. For indirect immunofluorescence, cells were plated in an 8-well Nunc Lab-Tek II chamber slides (ThermoFisher Scientific) and grown for 48 h. Cells were fixed with buffered 2% formaldehyde for 15 min at room temperature, and permeating in 0.2% Triton X-100 in PBS containing 1% BSA for 10 min at room temperature. Cells were incubated with both mouse anti-NME1 antibody (Abnova) and rabbit anti-Gelsolin antibody (Abcam) diluted in washing buffer at room temperature for 1 h, followed by incubation with goat anti-mouse antibody and goat anti-rabbit antibody (BioActs, Inchon, Korea). Nuclei were visualized by using 4’,6-diamidimo-2-phenylindole (DAPI: Sigma). Fluorescence imaging was acquired using a laser scanning confocal microscope (Zeiss LSM 780: Goettingen, Germany). Antibodies used including western blotting, IHC, immunoprecipitation, and indirect immunofluorescence are summarized in S4 Table.

### Cell proliferation and anoikis assays

Control and treated CRC cells were seeded onto 96-well plates to assess proliferation. Fold-changes in the number of cells were measured every day for 5 days using a cell proliferation assay kit (CCK-8; Dojindo, Kumamoto, Japan) on a microtiter plate reader adjusted to measure absorbance at 450 nm (Tecan, Melbourne, Australia). For the anoikis assay, 1 × 10^6^ cells were cultured on 6-well ultra-low attachment plates and normal culture plastic plates (Corning #3471 and #3516, Tewksbury, MA, USA) for 24 h. Suspended and adherent cells were harvested to measure apoptosis on a flow cytometer (Becton Dickinson, Franklin Lakes, NJ, USA) using Annexin V.

### Invasion assay and gelatin zymography

The transwell cell invasion assay measures both chemotaxis and invasion of cells through extracellular matrix [9]. Control and treated CRC cells (2 × 10^5^ cells each) were seeded onto the upper chamber of 24-well culture plates using a Biocoat™ Matrigel invasion chamber (BD Biosciences, San Jose, CA, USA). The 3T3-fibroblast-conditioned medium was placed in the lower chamber as a chemoattractant. After incubation at 37°C for 24 h, adherent cells were counted in three different fields under a light microscope (×100), and all assays were performed in triplicate. Matrix metalloprotease (MMP)-2 and MMP-9 activities in the culture media were examined by gelatine zymography as previously described [10].

### Statistical analysis

The demographic and biological features of patients with and without systemic recurrence were compared using Fisher’s exact test or an unpaired Student’s *t*-test. Differential mRNA expression and cellular activity were compared between the two groups using the Mann-Whitney *u*-test. An adequate survival analysis could not be performed in our training cohorts according to disproportionate tumor stages and treatment modality; therefore, the public database of the French Ligue Nationale Contre le Cancer (CIT cohort, GSE39582, n = 566) was used (S5 Table). To calculate the best cutoff for the expression of each gene, a receiver operating characteristics analysis was performed in which the optimal cutoff value was determined as the expression with the highest sensitivity and specificity. Recurrence-free survival (RFS) was compared using the Kaplan-Meier method with the log-rank test. Statistical significance was assigned when the *p*-values were <0.05. All calculations were performed using SPSS software (ver. 21, SPSS Inc., Chicago, IL, USA).

## Results

### Identification of six genes associated with LVI and PNI

The 117 recurrence-associated genes identified based on the cutoff of log_2_ TPM of mean values were compared according to LVI and PNI status in the initial 130 patients. LVI was closely associated with the expression of *OAS2* (*p* = 0.009), whereas PNI correlated with *GSN* (*p* = 0.029), *UDP1A6* (*p* = 0.004), *PALMD* (*p* = 0.003), *SNCG* (*p* = 0.004) and *HSPB6* (*p* = 0.002) (Table 1).

**Table 1:** Six selected genes associated with lymphovascular invasion or perineural invasion.

Genes	LVI, no of >mean mRNA expression				PNI, no of >mean mRNA expression			
	– vs. +	OR	95% CI	*p*-value^a^	– vs. +	OR	95% CI	*p*-value^a^
*UDP1A6*	34/85 vs. 17/45	0.911	0.433–1.914	0.852	45/96 vs. 6/34	0.243	0.092–0.64	0.004
*PALMD*	28/85 vs. 17/45	0.236	0.582–2.626	0.699	26/96 vs. 19/34	3.41	1.516–7.689	0.003
*SNCG*	31/85 vs. 18/45	1.161	0.553–2.439	0.708	29/96 vs. 20/34	3.3	1.468–7.42	0.004
*HSPB6*	30/85 vs. 16/45	1.011	0.475–2.153	1	26/96 vs. 20/34	3.846	1.697–8.715	0.002
*GSN*	39/85 vs. 20/45	0.944	0.456–1.951	1	38/96 vs. 21/34	2.466	1.104–5.507	0.029
*OAS2*	43/85 vs. 12/45	0.355	0.162–0.779	0.009	40/96 vs. 15/34	1.105	0.502–2.434	0.842

LVI, Lymphovascular invasion; PNI, perineural invasion; OR, odds ratio; CI, confidence interval.

^a^All parameters were compared using Fisher’s exact test with two-sided verification.

### GSN and OAS2 are implicated in CRC cell proliferation, anoikis, and invasion

**Fig 2:**
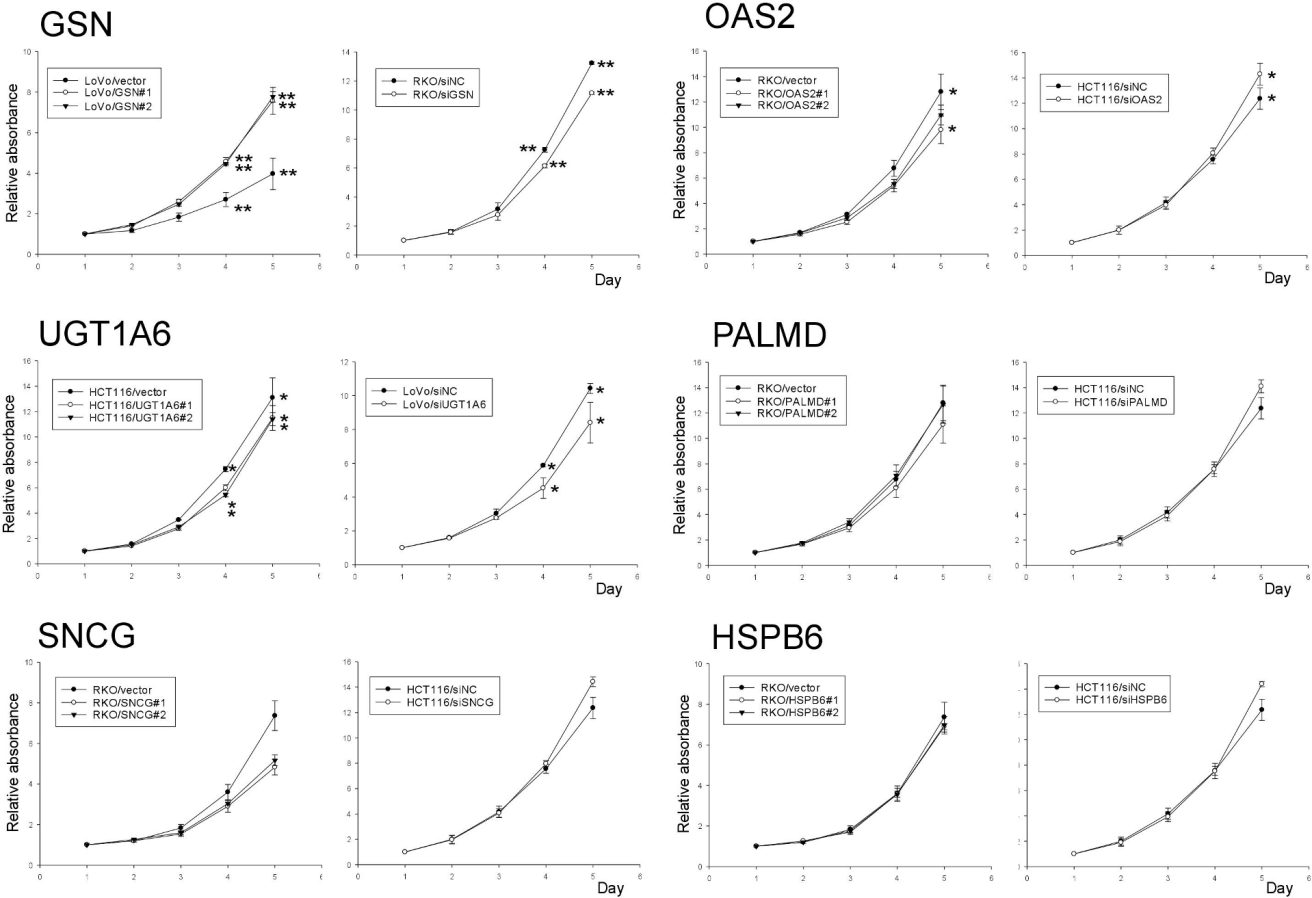
Proliferative activities of six molecules in overexpressing (left) and underexpressing (right) cells. SiNC, negative control siRNA. *p < 0.01–0.05; **p < 0.001.

**Fig 3:**
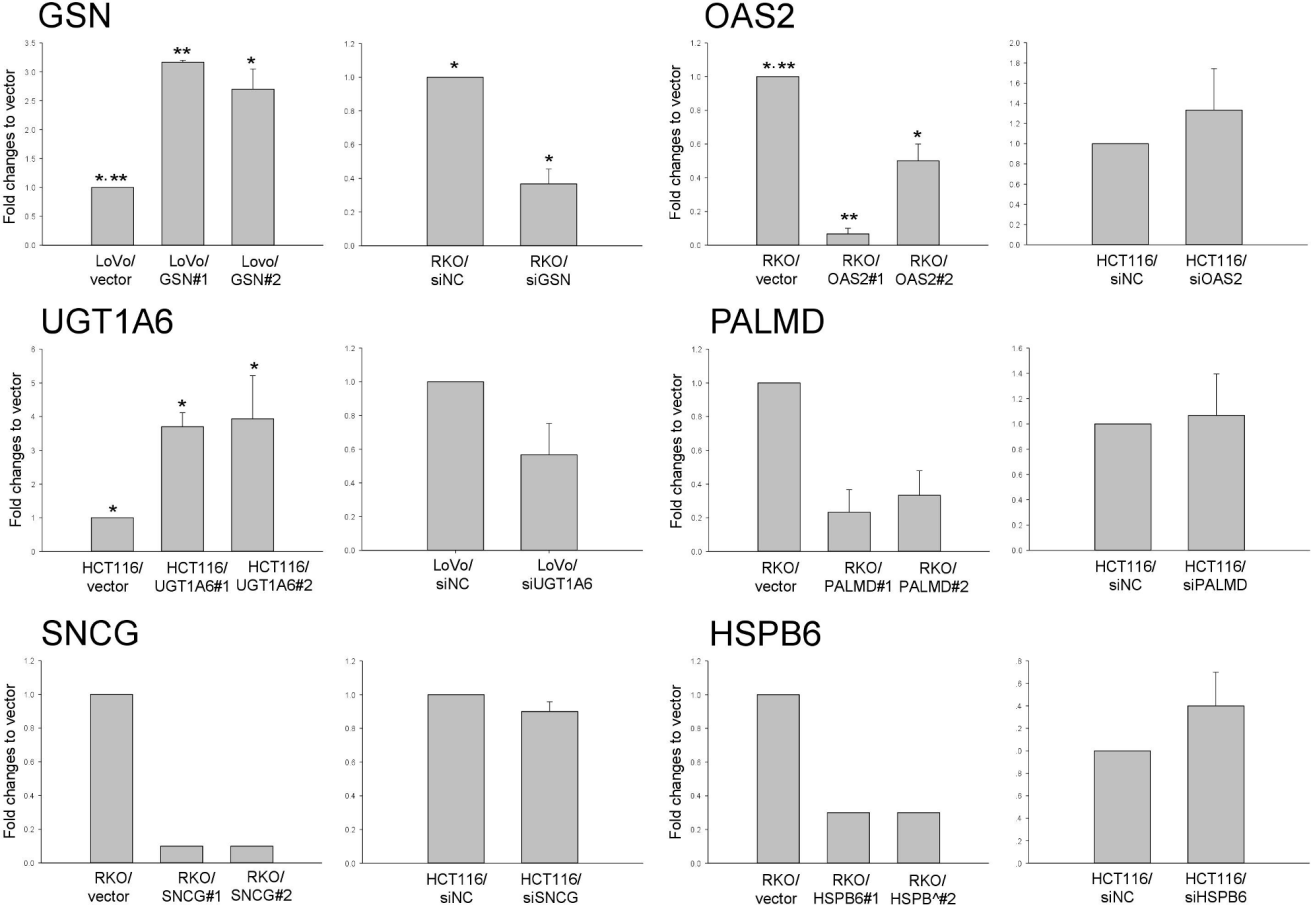
Invasive activities of six molecules in overexpressing (left) and underexpressing (right) cells. SiNC, negative control siRNA. *p < 0.01–0.05; **p < 0.001.

The proliferative activities of the six molecules identified were measured in the two clones of CRC cells overexpressing GSN or OAS2 (Fig 2). The proliferation of GSN-overexpressing LoVo cells increased significantly in a time-dependent manner between days 4 and 5 (*p* < 0.001) compared with that of control cells, whereas OAS2-overexpressing RKO cells and UGT1A6-overexpressing HCT116 cells showed reduced proliferation rates during the same period (*p* < 0.001–0.005). The opposite proliferation pattern was observed in GSN- and OAS2-underexpressing CRC cells. Anoikis resistance, which mediates the survival of cancer cells when they detach from the extracellular matrix and disseminate into the circulation, was examined next (S3 Fig). The relative apoptosis of suspended GSN-overexpressing cells decreased significantly by 40% at 0–24 h in GSN-overexpressing cells compared with that in control cells (*p* ≤ 0.001). The relative apoptosis rates did not differ between OAS2-overexpressing RKO cells and control cells. In invasion assays, GSN-overexpressing LoVo cells showed >2-fold greater invasiveness than control cells (*p* < 0.001–0.005), whereas OAS2-overexpressing RKO cells showed significantly reduced invasiveness (*p* < 0.001–0.005) (Fig 3). The opposite pattern was observed in GSN- and OAS2-underexpressing CRC cells. The remaining four molecules did not show significant differences in invasiveness between the corresponding overexpressing and control cells.

### Invasive nature of CRC cells mediated by GSN and OAS2

**Fig 4:**
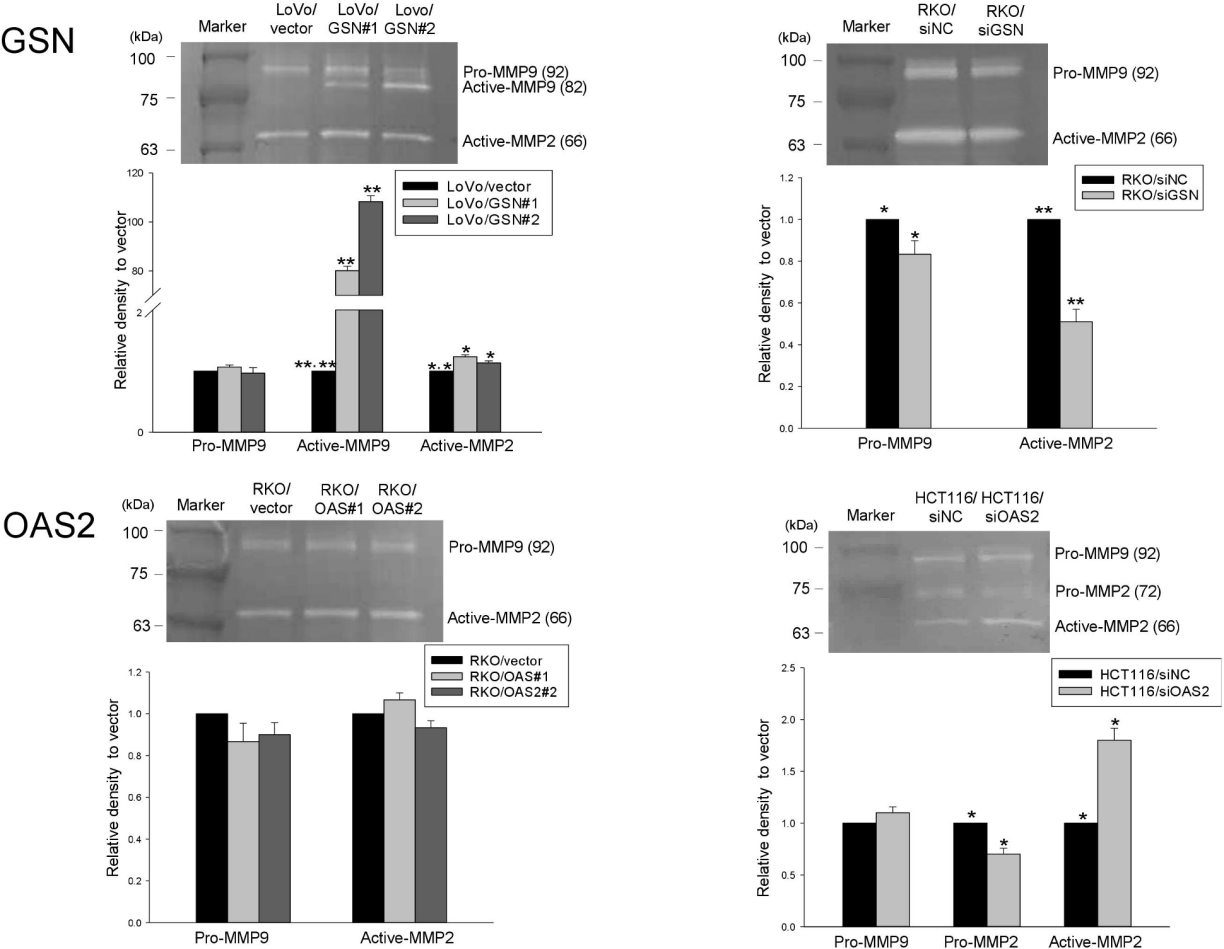
Invasive activities of GSN- and OAS2-overexpressing (left) and -underexpressing (right) cells measured by gelatine zymography. SiNC, negative control siRNA. *p < 0.01–0.05; **p < 0.001.

Invasive property of GSN and OAS2 was further assessed by gelatine zymography using MMP2 and MMP9 (Fig 4). GSN-overexpressing cells showed a marked increase of the active form of MMP9 (>80-fold, *p* < 0.001) and the active form of MMP2 (*p* < 0.05), whereas a reduced expression of active MMP2 was identified in GSN-underexpressing cells (*p* < 0.001). OAS2-underexpressing cells showed a significant increase in pro-MMP2 activity concurrent with an inversely proportional increase in the active form of MMP2 (*p* = 0.007 and 0.002, respectively). IHC analysis of a separate set of tumor tissues from 20 patients (10 patients each with and without recurrence) detected cytoplasmic expression of GSN and OAS2 (S6 Table). The invasive front (arrow) showed stronger GSN immunoreactivity than the central tumor in seven patients (35%; 4 patients with recurrence and 3 patients without recurrence), whereas OAS2 immunoreactivity was not different between invasive front and central tumor regardless of recurrence (S4 Fig).

### Colocalization of GSN and Nm23-H1

**Fig 5:**
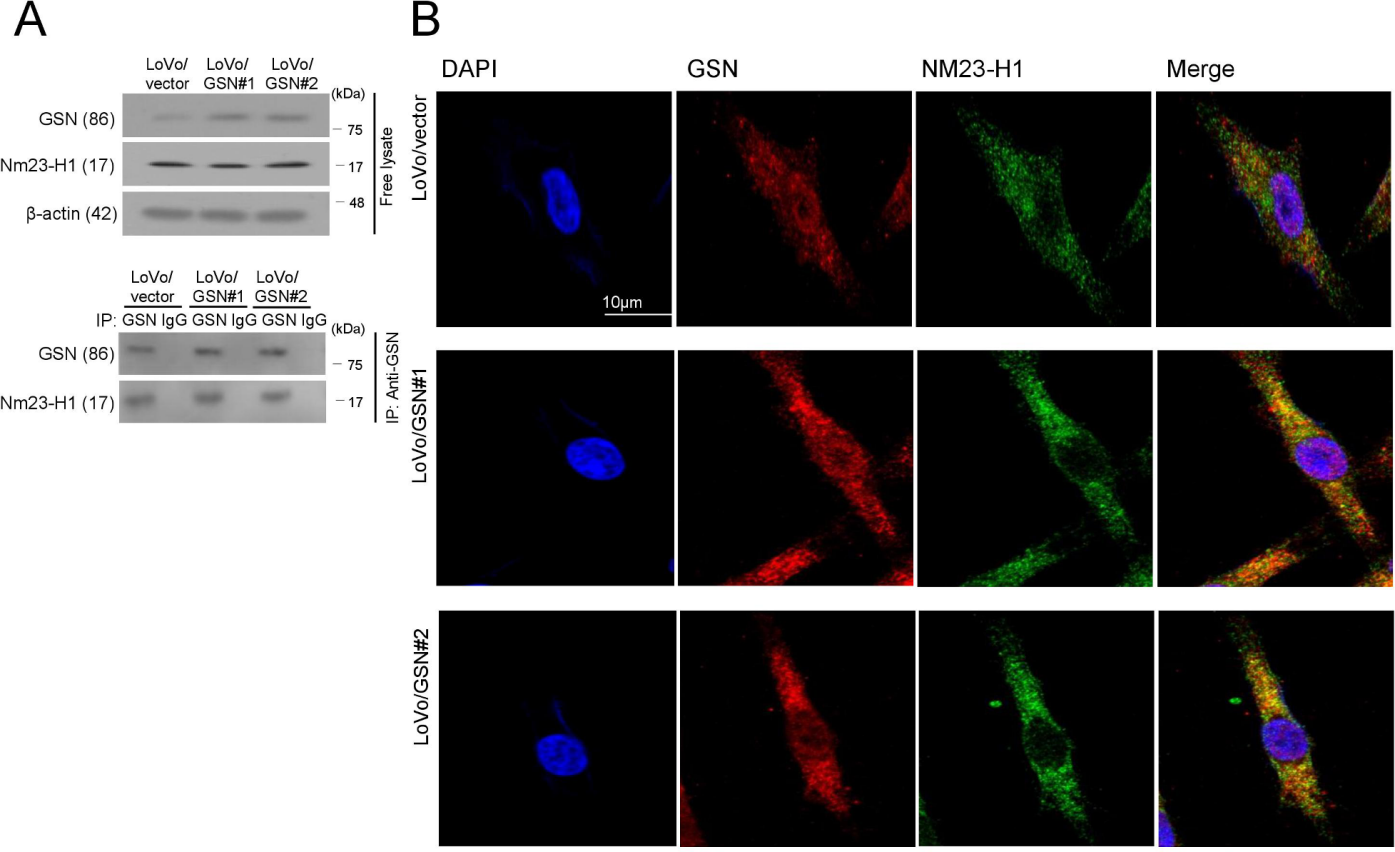
GSN immunoreactivity with Nm23-H1 was examined by immunoblotting/ immunoprecipitation (A) and indirect immunofluorescence (B). An anti-GSN monoclonal antibody was used to pull down the endogenous and overexpressed GSN. As GSN is exclusively observed in the cytoplasm, the GSN in this study indicates isoform 2.

The regulation of GSN activity was further examined using the Nm23-H1 gene by immunoblotting, immunoprecipitation and indirect immunofluorescence. An anti-GSN monoclonal antibody was used to pull down endogenous and overexpressed GSN. The Nm23-H1–GSN complex was detected in GSN-overexpressing cells and control cells by immunoprecipitation (Fig 5A). Immunofluorescence analysis confirmed the colocalization of the two proteins in the perinuclear and cytoplasmic compartments of GSN-overexpressing cells, whereas colocalization was minimal in control cells (Fig 5B).

### GSN and OAS2 are associated with epithelial-mesenchymal transition (EMT)

**Fig 6:**
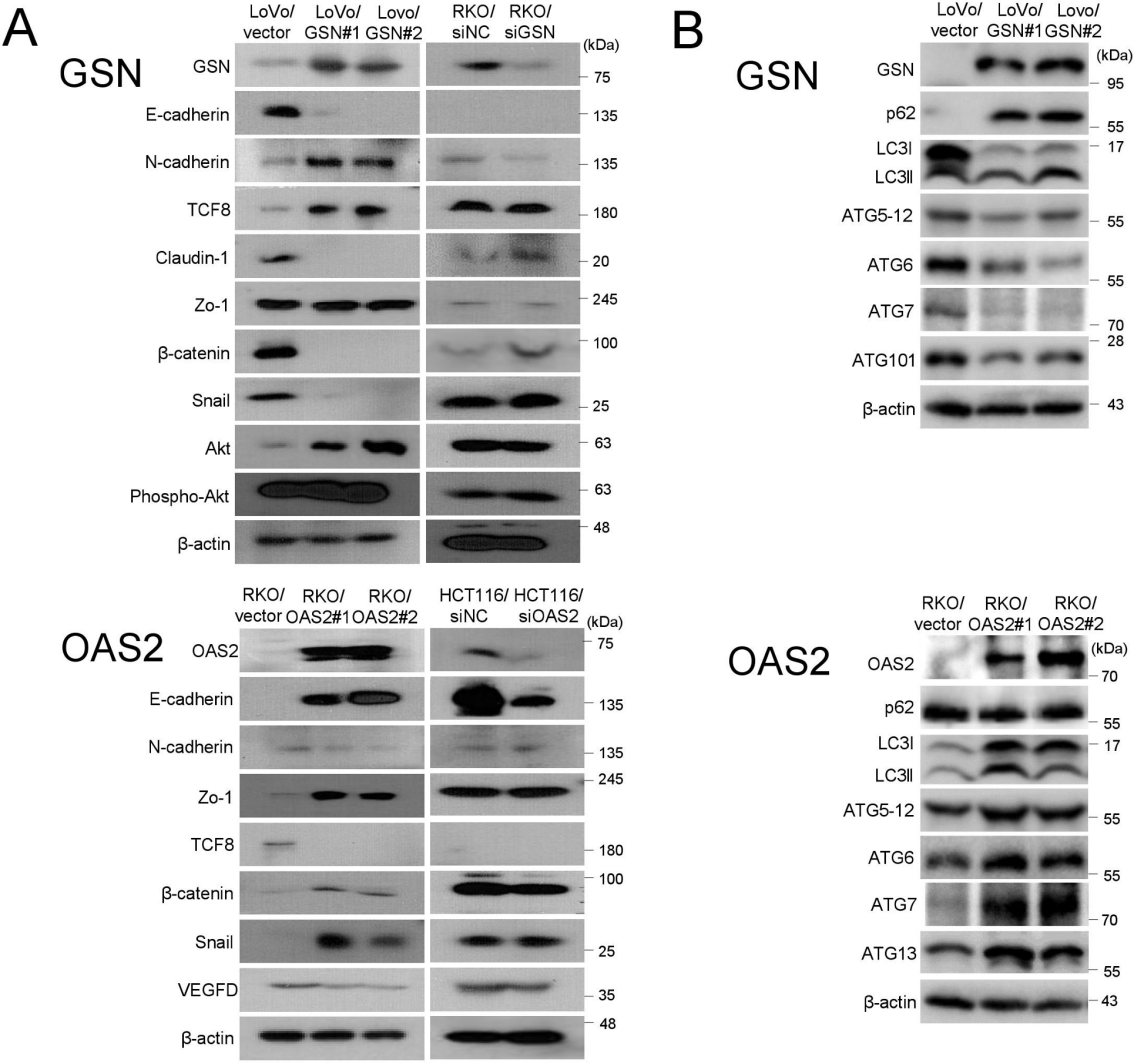
A. Immunoblotting of epithelial-mesenchymal transition (EMT) and pathway-related molecules in GSN- and OAS2-overexpressing (left columns) and -underexpressing (right columns) cells. B. Immunoblotting of autophagy-related molecules in GSN- and OAS2-overexpressing cells. SiNC, negative control siRNA.

The expression of EMT molecules was examined in GSN- and OAS2-overexpressing and -underexpressing CRC cells in comparison with vector-treated cells (Fig 6A). The expression of E-cadherin, β-catenin, claudin-1 and snail was lower, whereas that of N-cadherin and ZEB1 was higher, in GSN-overexpressing LoVo cells than in control cells. Phospho-Akt was concurrently increased in GSN-overexpressing LoVo cells compared with control cells. E-cadherin, β-catenin, Zo-1 and snail levels were higher in OAS2-overexpressing RKO cells than in control cells, whereas those of N-cadherin and ZEB1 were decreased. VEGFD expression was lower in OAS2-overexpressing RKO cells than in control cells. A reverse pattern was observed in GSN- and OAS2-underexpressing cells.

### GSN and OAS2 regulate expression of ATG molecules

Downregulation of BECN1 and LC3 is associated with metastasis of CRC cells [11]. To further elucidate the role of GSN and OAS2 in CRC metastasis, we examined the expression changes of autophagy-related genes that could be involved in the regulation of metastasis in GSN- and OAS2-overexpressing cells (Fig 6B). Several autophagy-related proteins including ATG5-12, ATG6/BECN1, ATG7 and ATG101 were downregulated in GSN-overexpressing LoVo cells. In addition, the accumulation of p62, a substrate of autophagic degradation [12], suggested that autophagic flux was reduced in GSN-overexpressing LoVo cells. In contrast to the pattern in GSN-overexpressing cells, the levels of ATG5-12, ATG6, ATG7 and LC3II accumulation were increased in OAS2-overexpressing RKO cells, indicating that ectopic expression of OAS2 promoted autophagy activation in CRC cells.

### GSN and OAS2 mRNA expressions predict disease-free survival

**Fig 7:**
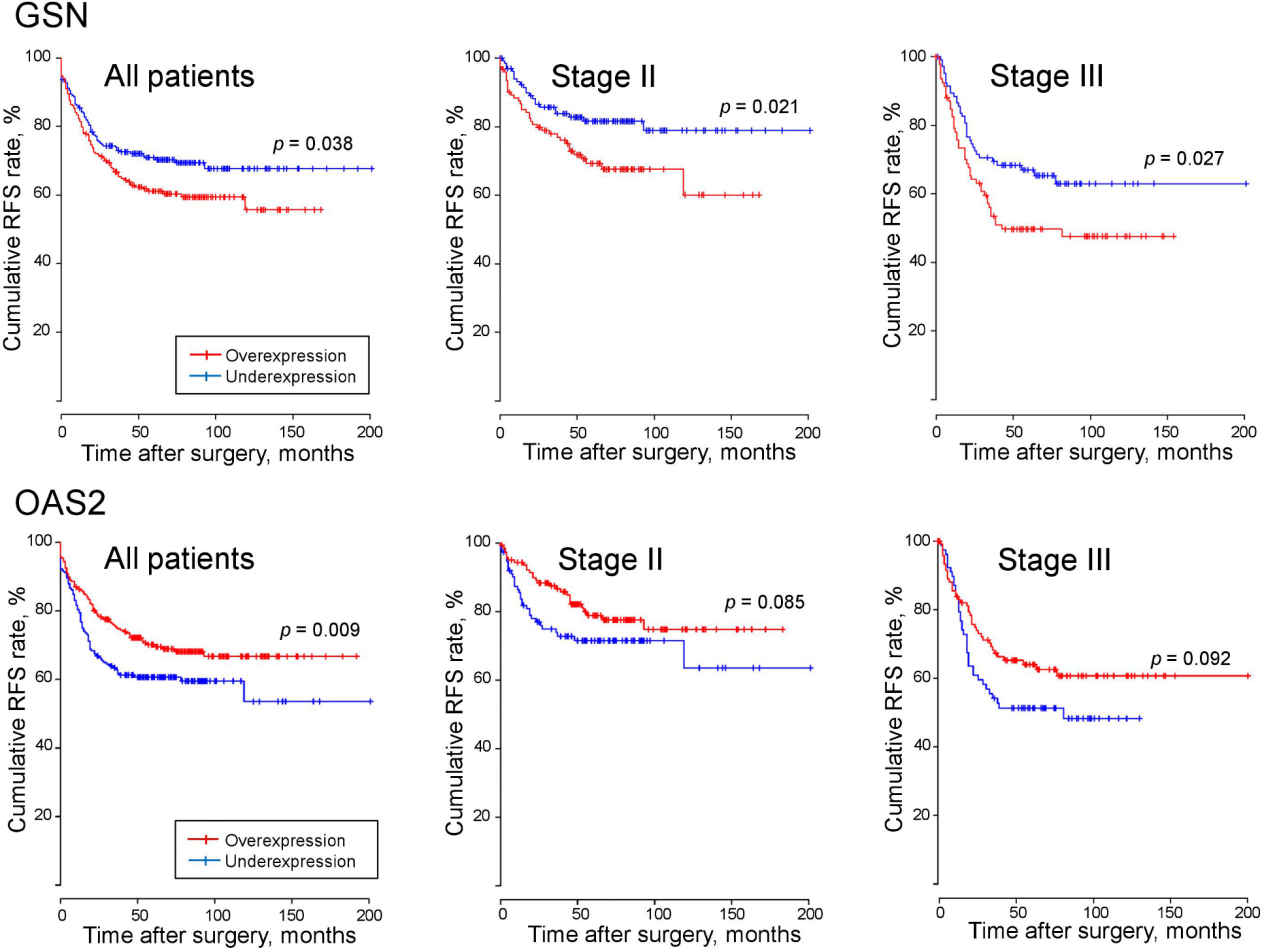
Recurrence-free survival (RFS) was compared between GSN and OAS2 mRNA-overexpressing and–underexpressing tumors in the CIT cohorts (GSE39582, n = 566).

The value of GSN and OAS2 mRNA expression for predicting recurrence was determined by examining RFS rates in the CIT cohort (Fig 7). The CRC samples were divided into two groups according to the cutoff values representing the maximal level of sensitivity multiplied by specificity (GSN = 4.5182 and OAS2 = 3.3402). The 5-year RFS rates were significantly greater in the GSN underexpression group than in the overexpression group (73.6% vs. 64.7%, *p* = 0.038), whereas patients with OAS2 overexpression had a greater RFS rate than those with underexpression (73.4% vs. 63.7%, *p* = 0.01). Survival outcomes according to tumor stage confirmed significant differences between the two groups of GSN expression (*p* = 0.021 for stage II patients; *p* = 0.027 for stage III patients), and showed a trend toward better survival rates in the OAS2 overexpression group than in the underexpression group.

## Discussion

A total of 117 genes differentially expressed between CRC patients with and without systemic recurrence were initially selected, minimising false discovery. These gene sets were further narrowed down to six genes according to the criteria of their correlations with LVI and PNI. The findings of proliferation and invasion assays led to the selection of GSN and OAS2 as potential molecules related with systemic recurrence of CRC via PNI and LVI, respectively.

In the present study, GSN strongly promoted cellular proliferation and invasion in GSN-overexpressing cells, whereas the opposite pattern was observed in underexpressing cells. GSN is an actin-modulating protein with diverse biological functions associated with tumorigenesis and progression, including differentiation, apoptosis, proliferation, invasion and migration [13]. However, GSN has a dual function as tumor suppressor or promoter [14,15]. Gelatine zymography is used to measure MMP activity, particularly that of the active forms of MMP2 and MMP9, which play a crucial role in the degradation of the basement membrane and extracellular matrix [16,17]. We consistently found a marked upregulation of active MMP9 and a partial increase of active MMP2. GSN immunoreactivity was more prominent at the invasive front than in the central tumor in approximately 1/3 of tissue samples, indicating individual susceptibility to GSN-mediated invasion.

We further examined the regulatory molecules involved in GSN activation. Nm23 inhibits molecules and signalling pathways associated with tumor invasion such as MMP-2 and the MAPK and TGF-β pathways [17,18]; however, the protein interactions involved remain unclear. A recent study demonstrated that Nm23-H1 inhibits the motility-promoting effects of GSN, consistent with its abrogation of actin-severing abilities [15]. We found a strong colocalization of the two proteins in the perinuclear and cytoplasmic compartments of GSN-overexpressing cells, whereas minimal colocalization was detected in control cells. Taken together, these findings suggest that PNI-associated GSN may promote chemotactic invasion by interacting with Nm23-H1, maintaining anoikis resistance.

OAS2 overexpression is reported in patients with inflammatory, autoimmune and malignant diseases, although many of its biological functions remain to be clarified [19,20]. In the present study, OAS2-overexpressing cells consistently showed reduced proliferation and invasion compared with those in control cells, whereas OAS2 underexpressing cells showed the opposite pattern. A previous study using murine pancreatic β-cells reported that OAS2 overexpression inhibited cell proliferation [21].

Epithelial cancer cells undergoing EMT have an invasive or metastatic phenotype [22]. Loss of E-cadherin expression is a key event during EMT in contrast with proteasome-mediated degradation of β-catenin [23,24]. In the present study, GSN downregulated E-cadherin, β-catenin, claudin-1 and snail, whereas it upregulated N-cadherin and ZEB1, and OAS2 had the opposite effects. Tumor cells showing a cadherin switch (loss of E-cadherin and gain of N-cadherin expression) exhibit aggressive metastatic phenotypes [25]. Loss of the tight-junction protein claudin-1 is associated with aggressive cancer behavior, deeper tumor invasion, advanced tumor grade, lymph node metastasis, PNI, LVI and recurrence [26]. E-cadherin underexpression and ZEB1 overexpression are correlated with poor survival in CRC patients [23]. Additionally, we found that phospho-Akt was upregulated in GSN-overexpressing cells compared with control cells. Activation of PI3K/Akt signalling represses E-cadherin transcription by stabilising transcriptional repressors including snail and slug, promoting growth and progression of CRC [27]. In the lymphangiogenic pathway, the VEGFC–VEGFR3 and VEGFD–VEGFR3 axes are required for the dissemination of cancer cells to systemic lymph nodes and distant organs [28]. VEGFD expression was significantly reduced in OAS2-overexpressing cells, and VEGFD is an independent poor prognostic indicator in CRC patients [29].

Accumulating evidence indicates that autophagy defects are closely correlated with malignant phenotypes such as metastasis and poor prognosis in various cancer cells [30,31]. Consistent with these results, we found that overexpression of GSN downregulated several autophagy-related proteins and suppressed autophagy activation. A recent study showed that overexpression of GSN increases the levels of reactive oxygen species (ROS) by inhibiting Cu/Zn-superoxide dismutase activity [32]. In addition, downregulation of autophagy-related genes promotes metastasis by inducing ROS production and HIF-1α expression [31,33]. In the present study, overexpression of OAS2 promoted autophagy, as demonstrated by the upregulation of a subset of autophagy genes. Extensive evidence supports the role of autophagy in the regulation of immune responses and cancer immunotherapy [34], whereas the role of OAS2 in autophagy remains unknown. Taken together, the present findings suggest that GSN and OAS2 repress and promote autophagy, respectively, and partly contribute to CRC progression and metastasis.

The 5-year DFS rates in the CIT cohort were significantly greater in the GSN-underexpressing and OAS2-overexpressing groups than in the opposite groups. A better survival outcome was also confirmed in the GSN-underexpressing group, as demonstrated by the respective stages (II and III). There are few reports on survival outcomes associated with GSN overexpression, although poor survival was reported in non-small cell lung cancer and osteosarcoma [35,36].

In conclusion, we showed that PNI-associated GSN induces cell proliferation and migration by promoting invasion into the extracellular matrix, whereas LVI-associated OAS2 suppresses these biological activities. The opposite functions of these two molecules may be mediated by EMT and autophagy flux. GSN and OAS2 were poor and favorable prognostic factors, respectively, in CRC patients. Although these findings were derived from strict criteria in terms of gene selection and functional validation, the present study had limitations that may affect the conclusions reached. Four genes that may be associated with recurrence were not included because they did not possess sufficient proliferative and invasive properties. In addition, survival outcome could not be analyzed in our cohort because of the heterogeneous population in terms of recurrence and treatment. Nevertheless, the present results suggest the potential value of GSN and OAS2 as predictors of recurrence or therapeutic targets, and these findings should be validated in the future in clinical studies.

## Supporting information

S1 Fig The mRNA expression of the six selected genes in two colorectal epithelial and 10 CRC cell lines. (TIF)

S2 Fig Stable expression of transfected molecules was evaluated by western blot analysis (left, cDNA transfection; right, siRNA transfection) in various colorectal cancer cells. SiNC, negative control siRNA.
(TIF)

S3 Fig Anoikis of suspended and adherent cells evaluated by measuring apoptosis using flow cytometry. The percentage of apoptotic cells was calculated as the sum of Annexin V- positive/propidium iodide-negative cells (early stages of apoptosis = Q4) and that of Annexin V-positive/propidium iodide-positive cells (late stages of apoptosis = Q2). SiNC, negative control siRNA. *p < 0.01–0.05; **p ≤ 0.001.
(TIF)

S4 Fig Immunohistochemistry analysis of the cytoplasmic expression of GSN (A and C) and OAS2 (B and D) in tumor tissues. Invading small glands (arrows) at the invasive front show stronger GSN immunoreactivity (A) and weaker OAS2 immunoreactivity (B) than the central tumor. Moderate GSN immunoreactivity in tumor cells surrounding the neural plexus (arrow, C) and diffuse weak OAS2 immunoreactivity between tumor cells invading lymphatics (D, arrow).
(TIF)

S1 Table Patient clinicopathological features. (DOCX)

S2 Table A total of 963 genes differentially expressed between patients with and without systemic recurrence. (XLSX)

S3 Table Primers for real time RT-PCR and siRNA sequences of 6 selected genes. (DOCX)

S4 Table Antibodies and methods of western blotting, immunohistochemistry, immunoprecipitation, and indirect immunofluorescence. (DOCX)

S5 Table Baseline characteristics of patients with colorectal cancer in the CIT cohort. (DOCX)

S6 Table Another cohort of 20 patients for immunohistochemical evaluation. (DOCX)

## Abbreviations

CRC: colorectal cancer
LVI: lymphovascular invasion
PNI: perineural invasion
AJCC: American joint committee on cancer
CDKN2A: cyclin-dependent kinase Inhibitor 2A
GLM: generalized linear model
FDR: false discovery rate
TPM: transcripts per million
GSN: gelsolin
OAS2: 2'-5'-oligoadenylate synthetase 2
UGT1A6: UDP glucuronosyltransferase family 1 member A6
PALMD: palmdelphin
SNCG: synuclein gamma
HSPB6: heat shock protein family B member 6
IHC: immunohistochemistry
MMP: matrix metalloproteinase
Nm23: nucleoside diphosphate kinase 1
EMT: epithelial mesenchymal transition
ZEB1: zinc finger E-box binding homeobox 1
Zo-1: zonula occludens-1
VEGF: vascular endothelial growth factor
VEGFR: vascular endothelial growth factor receptor
ROS: reactive oxygen species
