# Supplementary figures and images for: Opposite functions of GSN and OAS2 on colorectal cancer metastasis, mediating perineural and lymphovascular invasion, respectively
Source: PLoS One. 2018 Aug 27;13(8):e0202856. doi: 10.1371/journal.pone.0202856 (PMC6110496; doi:10.1371/journal.pone.0202856)

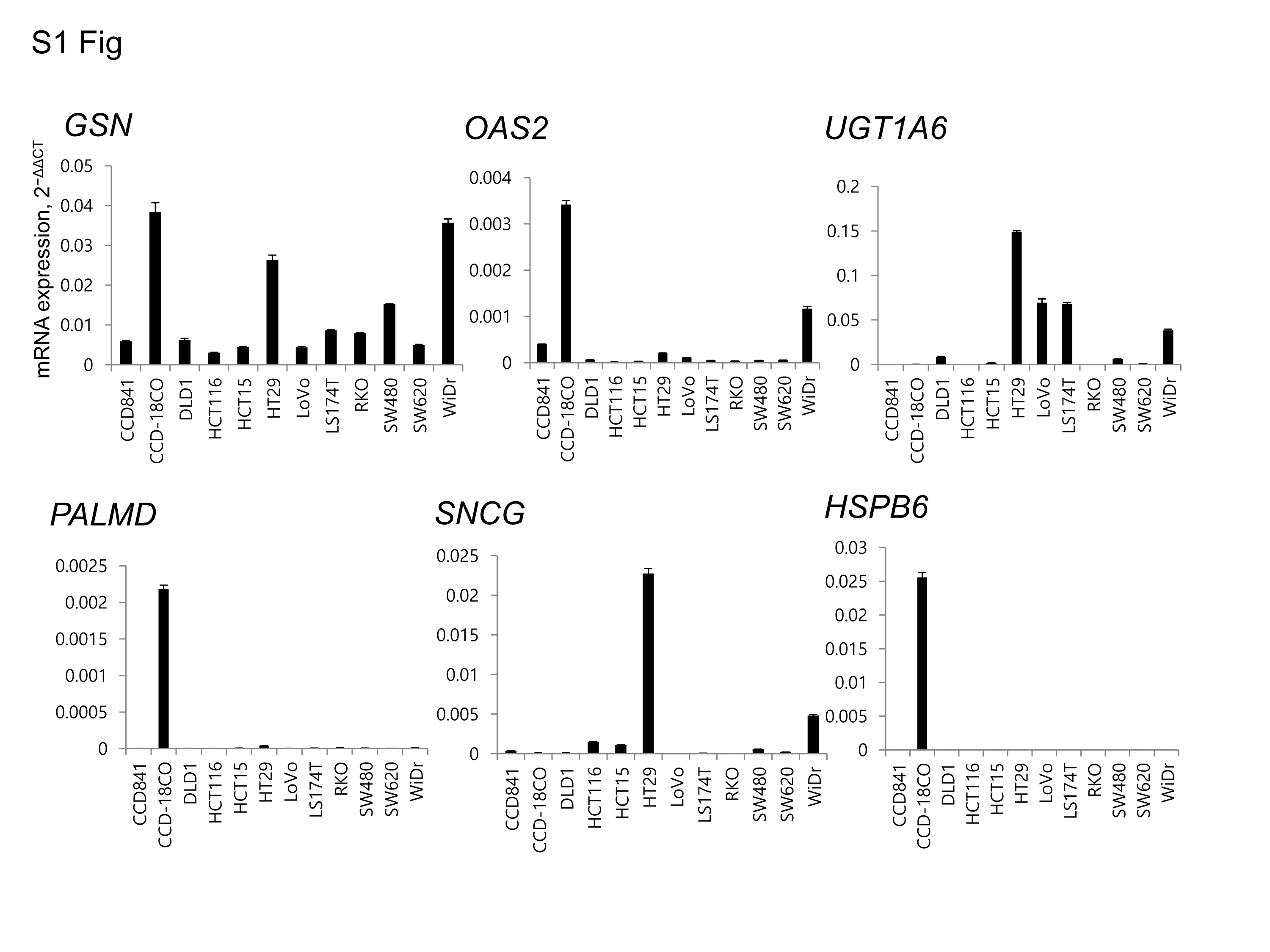

Supplement: S1 Fig — (TIF) [file pone.0202856.s001.tif]

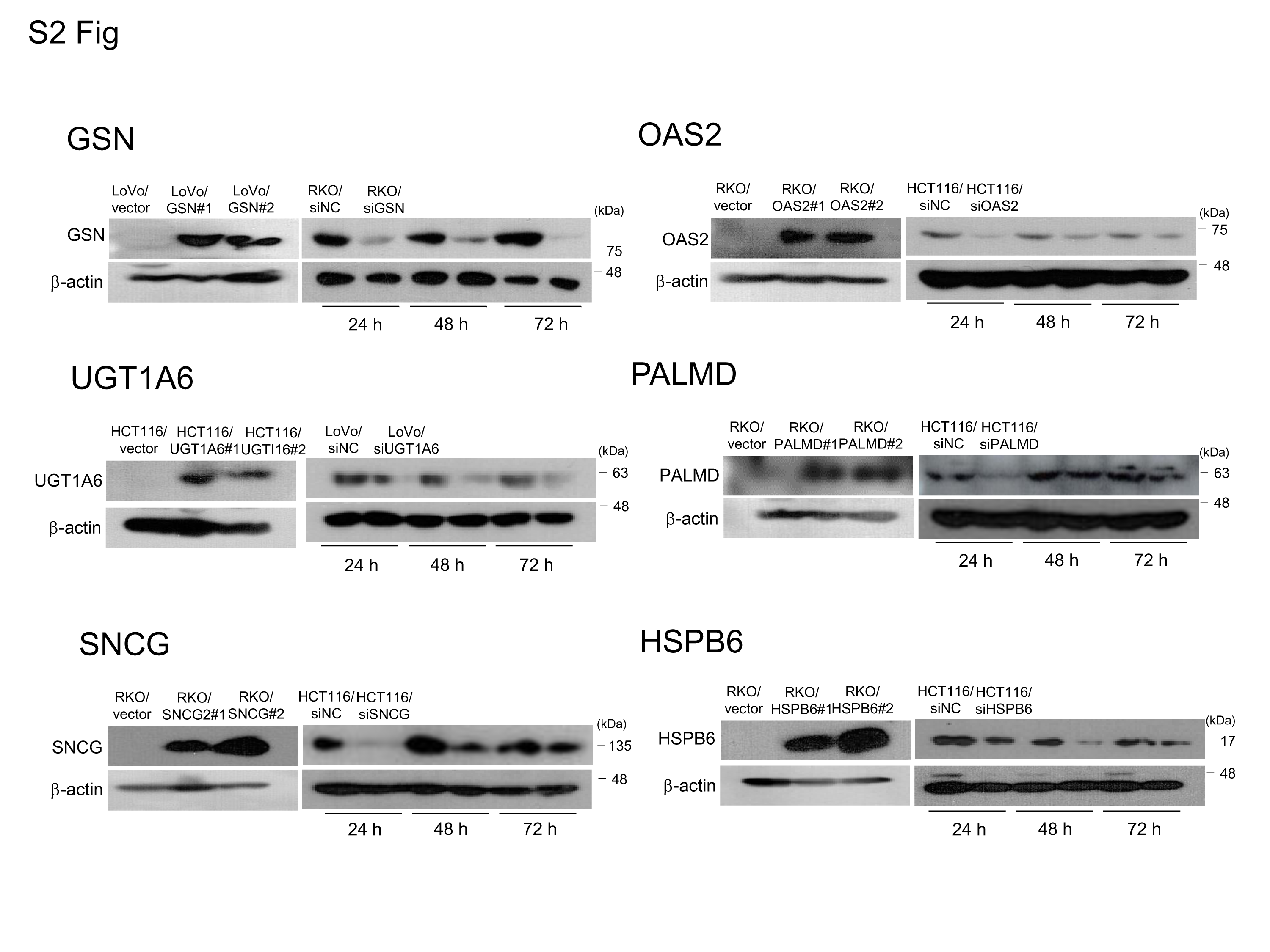

Supplement: S2 Fig — SiNC, negative control siRNA. (TIF) [file pone.0202856.s002.tif]

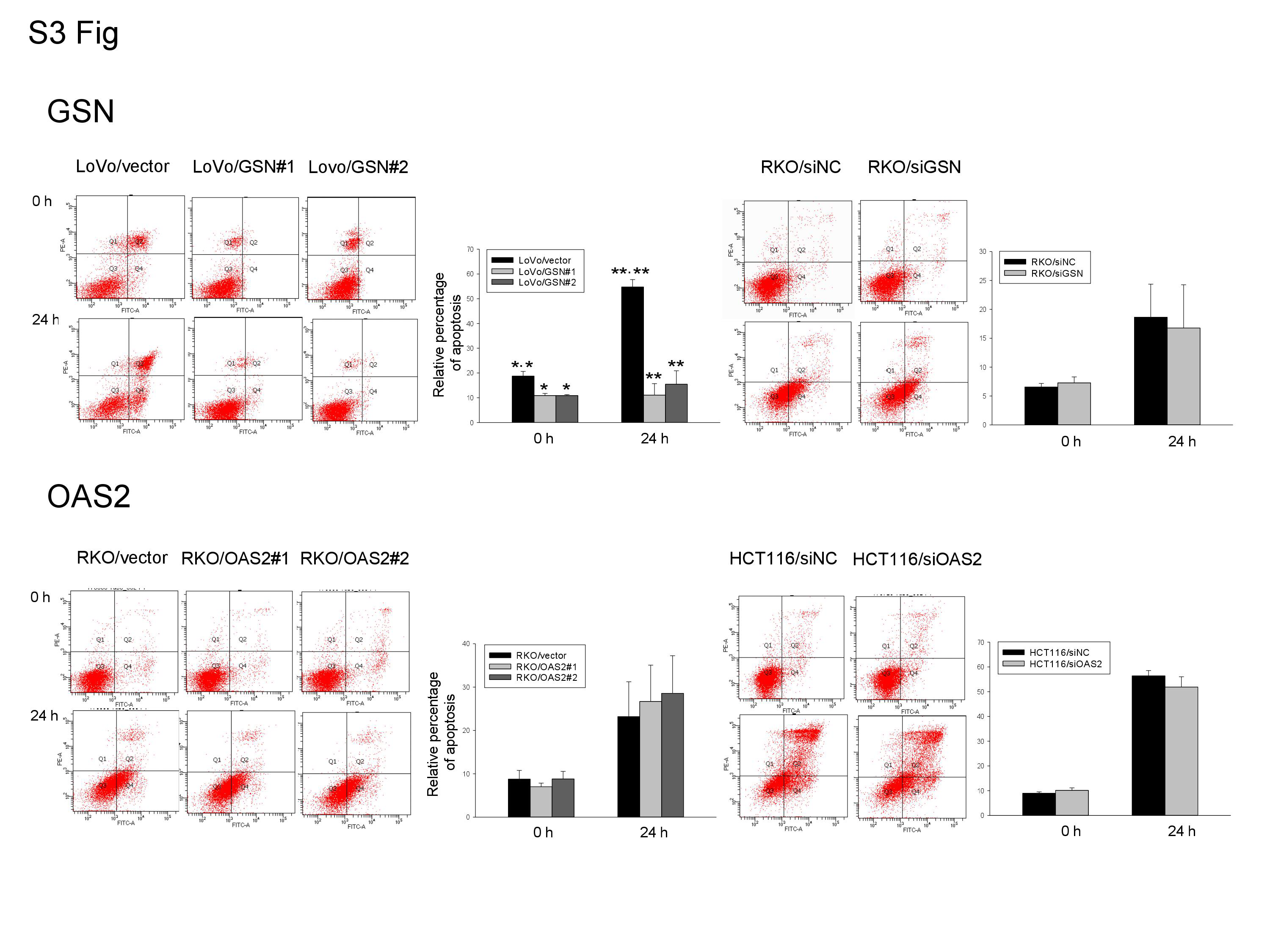

Supplement: S3 Fig — The percentage of apoptotic cells was calculated as the sum of Annexin V- positive/propidium iodide-negative cells (early stages of apoptosis = Q4) and that of Annexin V-positive/propidium iodide-positive cells (late stages of apoptosis = Q2). SiNC, negative control siRNA. *p < 0.01–0.05; **p ≤ 0.001. (TIF) [file pone.0202856.s003.tif]

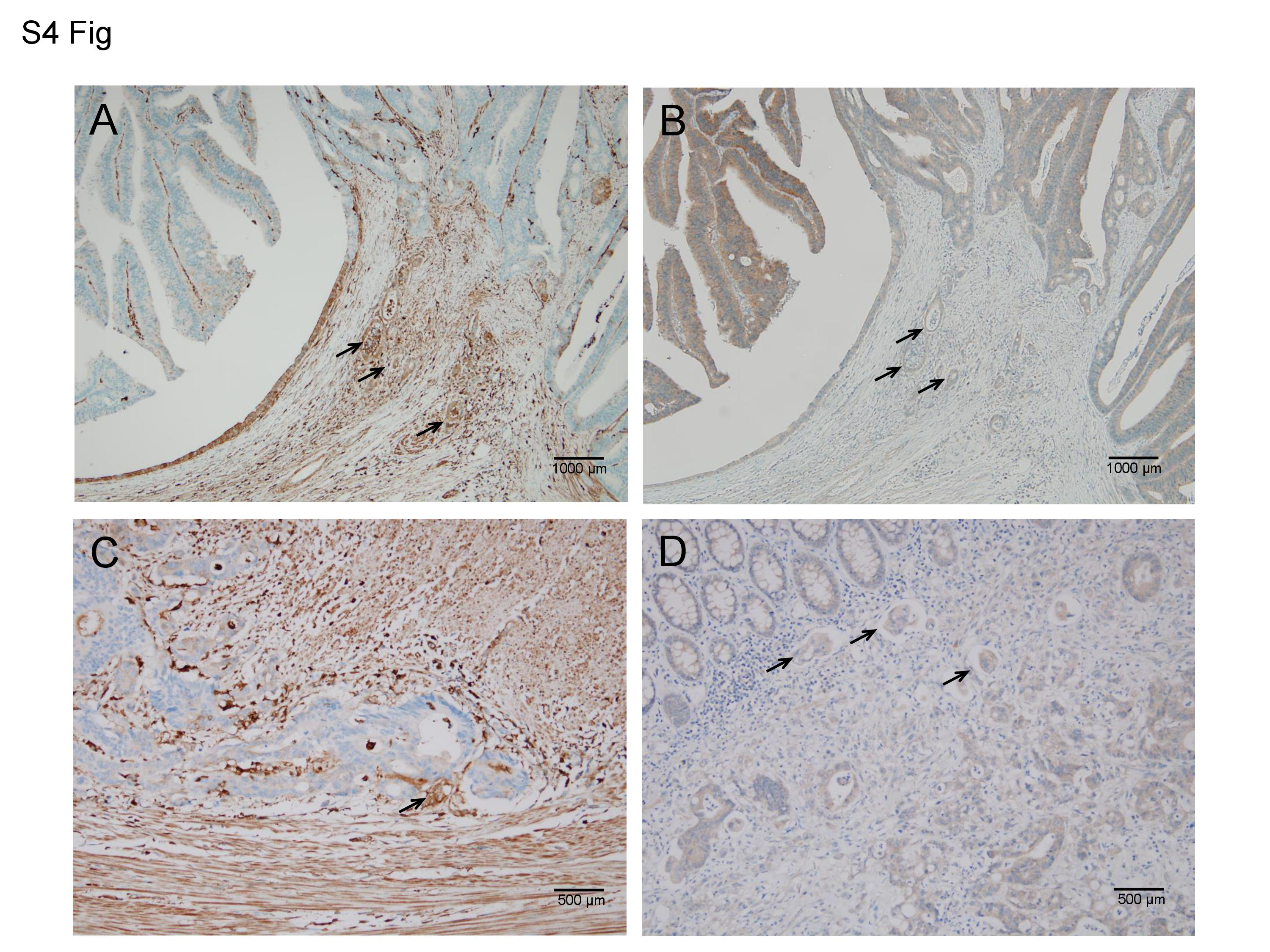

Supplement: S4 Fig — Immunohistochemistry analysis of the cytoplasmic expression of GSN (A and C) and OAS2 (B and D) in tumor tissues. Invading small glands (arrows) at the invasive front show stronger GSN immunoreactivity (A) and weaker OAS2 immunoreactivity (B) than the central tumor. Moderate GSN immunoreactivity in tumor cells surrounding the neural plexus (arrow, C) and diffuse weak OAS2 immunoreactivity between tumor cells invading lymphatics (D, arrow). (TIF) [file pone.0202856.s004.tif]
