# Supplementary material for: Opposite functions of GSN and OAS2 on colorectal cancer metastasis, mediating perineural and lymphovascular invasion, respectively
Source: PLoS One. 2018 Aug 27;13(8):e0202856. doi: 10.1371/journal.pone.0202856 (PMC6110496; doi:10.1371/journal.pone.0202856)
Supplement: S1 Table — (DOCX) [file pone.0202856.s005.docx]

**S1 Table. Patient clinicopathological features**

| Parameters^a^ | Systemic recurrence−, n = 72 | Systemic recurrence+, n = 58 | *p*^b^ | |
| --- | --- | --- | --- | --- |
| Sex, male/female | 40/32 | 39/19 | | 0.208 |
| Age, year | 58 ± 11 | 59 ± 11 | | 0.401 |
| Pathological stage^c^, 0/I/II/III/IV | 1/6/44/21/0 | 0/0/2/7/49 | | <0.001 |
| Recurrence, syn-/meta-chronous | N.A. | 49/9 | | N.A. |
| Primary tumour: |  |  | |  |
| Location^d^, right/left/rectum | 51/3/18 | 32/10/16 | | 0.033 |
| Growth, expanding/infiltrative | 54/18 | 47/11 | | 0.526 |
| Differentiation, W/M/P/mucinous | 2/63/4/3 | 2/51/2/3 | | 0.935 |
| Lymphovascular invasion, no/yes | 59/13 | 26/32 | | <0.001 |
| Perineural invasion, no/yes | 60/12 | 36/22 | | 0.009 |

N.A., not applicable; W/M/P, well-/moderately-/poorly-differentiated.

^a^Continuous values are means ± standard deviation; values in parentheses are percentages.

^b^All parameters were compared using Pearson’s χ^2^ and unpaired *t* tests.

^c^Pathological cancer staging according to the American joint committee on cancer (8th ed., 2017).

^d^Right, cecum – transverse colon; left, descending colon – sigmoid colon.
