# Supplementary material for: Opposite functions of GSN and OAS2 on colorectal cancer metastasis, mediating perineural and lymphovascular invasion, respectively
Source: PLoS One. 2018 Aug 27;13(8):e0202856. doi: 10.1371/journal.pone.0202856 (PMC6110496; doi:10.1371/journal.pone.0202856)
Supplement: S3 Table — (DOCX) [file pone.0202856.s007.docx]

**Table S3. Primers for real time RT-PCR and siRNA sequences of 6 selected genes**

| Genes | Primer and target sequence 5'-3' | RefSeq | Product (bp) | Tm, °C |
| --- | --- | --- | --- | --- |
| *OAS2* | Forward: GCTCCTATGGACGGAAAACA  Reverse: CAAGGGACTTCTGGATCTCG | NM_001032731.1 | 192 | 60 |
|  | siRNA: AGAGGCAACUCCGAUGGUA, AAGAGAAGCCAACGUGACA,  GGGAUAAGCUGAAGUUCUG, GUUGGUUUAUCCAGGAAUA |  |  |  |
| *UGT1A6* | Forward: GCAAAGCGCATGGAGACTAAGG  Reverse: GGTCCTTGTGAAGGCTGGAGAG | NM_001072 | 148 | 60 |
|  | siRNA: GGAGGUAUCAACUGUAAGA, GCGAACAACACGAUACUUG,  CGUGAUUGGUUUCCUCUUG, CAAAGCGCAUGGAGACUAA |  |  |  |
| *PALMD* | Forward: GAGGAAGACAAACTAAAGCACCAG  Reverse: CTCTTCCTGTTCTTTTCCGCTGC | NM_017734 | 97 | 60 |
|  | siRNA: GAUGGAAUCAGCAGCGGAA, GGGUAUUGGUGUAAAUGAA,  GGACAACAGAAGACAUUAU, AGGAAGAUGUCAGAUAUAA |  |  |  |
| *SNCG* | Forward: TGTGGTGAGCAGCGTCAACACT  Reverse: TTGGATGCCTCACCCTCCTGTT | NM_003087 | 131 | 60 |
|  | siRNA: CCAAGGAGAAUGUUGUACA, GCGGAGAACAUCGCGGUCA,  UGAGCAGCGUCAACACUGU, GAGACUAGAGGGCUACAGG |  |  |  |
| *HSPB6* | Forward: GCCACTTTTCGGTGCTGCTAGA  Reverse: GCGCGACGAATCCGTGCTCAT | NM_144617 | 130 | 60 |
|  | siRNA: CUACAAAGACAUCCGGGUA, CGGAGGAAAUUGCUGUCAA,  AACCAGAUAUCCUCGGCAA, CCUACCAGCACUACCCUAA |  |  |  |
| *GSN* | Forward: ATCTGCCATCCTGACTGCTCAG  Reverse: CTTCCCACCAAACAGGCTCATG | NM_000177 | 112 | 60 |
|  | siRNA: CCUGCUCCAACAAGAUUGGACGUUU |  |  |  |
