# Supplementary material for: Opposite functions of GSN and OAS2 on colorectal cancer metastasis, mediating perineural and lymphovascular invasion, respectively
Source: PLoS One. 2018 Aug 27;13(8):e0202856. doi: 10.1371/journal.pone.0202856 (PMC6110496; doi:10.1371/journal.pone.0202856)
Supplement: S4 Table — (DOCX) [file pone.0202856.s008.docx]

**Table S4. Antibodies and methods of western blotting, immunohistochemistry, Immunoprecipitation, and indirect immunofluorescence**

| Antibodies | Products |
| --- | --- |
| Anti-OAS2, Anti-HSPB6, Anti-GSN, Anti-PALMD, Anti-SNCG, Anti-UGT1A6 Antibodies, Anti-ATG7, Anti-ATG13, Anti-ATG16L, Anti-ATG101 | Abcam, Cambridge, UK |
| Anti-ATG4B, Anti-p62, Anti-LC3, Anti-BECN1, Anti-DYKDDDDK, Anti-ATG14 | Cell Signaling Technology, Beverly, MA, USA |
| Anti-ATG5 | Biologicals, Littleton, CO, USA |
| Anti-ATG10 | Woburn, MA, USA |
| Anti-Actin | Millipore, Temecula, CA, USA |
| EMT antibody sampler kit | Cell Signaling, Beverly, MA, USA |
| HRP-conjugated secondary antibody | Pierce, Rockford, IL, USA |
