# Supplementary material for: Opposite functions of GSN and OAS2 on colorectal cancer metastasis, mediating perineural and lymphovascular invasion, respectively
Source: PLoS One. 2018 Aug 27;13(8):e0202856. doi: 10.1371/journal.pone.0202856 (PMC6110496; doi:10.1371/journal.pone.0202856)
Supplement: S5 Table — (DOCX) [file pone.0202856.s009.docx]

**Table S5. Baseline characteristics of patients with colorectal cancer in the CIT cohort**

| Parameters | No of patients (%), total no=566 |
| --- | --- |
| Gender, male/female | 310/256 (54.8/45.2) |
| Median age, range, y | 68, 22-97 |
| Location, colon/rectum | 566/0 (100/0) |
| AJCC stage, I/II/III/IV | 37/264/205/60 (6.5/46.7/36.2/10.6) |
| Adjuvant chemotherapy, yes/no/not available | 233/316/17 (41.2/55.8/3) |
| No of events | 177 |
| Median disease-free survival, m | 48 |

CIT, Cartes d'Identité des Tumeurs ; AJCC, American joint committee on cancer.
