# Supplementary material for: Opposite functions of GSN and OAS2 on colorectal cancer metastasis, mediating perineural and lymphovascular invasion, respectively
Source: PLoS One. 2018 Aug 27;13(8):e0202856. doi: 10.1371/journal.pone.0202856 (PMC6110496; doi:10.1371/journal.pone.0202856)
Supplement: S6 Table — (DOCX) [file pone.0202856.s010.docx]

**Table S6. Another cohort of 20 patients for immunohistochemical evaluation**

| Parameters^a^ | Systemic recurrence−, n = 10 | Systemic recurrence+, n = 10 | *p*^b^ | |
| --- | --- | --- | --- | --- |
| Sex, male/female | 2/8 | 1/9 | | 1 |
| Age, year | 59 ± 7 | 56 ± 7 | | 0.406 |
| Pathological stage^c^, 0/I/II/III/IV | 1/4/5/0 | 0/4/3/3 | | 0.212 |
| Primary tumour: |  |  | |  |
| Location^d^, right/left/rectum | 6/3/1 | 3/2/5 | | 0.145 |
| Growth, expanding/infiltrative | 9/1 | 8/2 | | 1 |
| Differentiation, W/M/P/mucinous | 0/9/0/1 | 0/7/2/1 | | 0.325 |
| Lymphovascuar invasion, no/yes | 10/0 | 4/6 | | 0.011 |
| Perineural invasion, no/yes | 8/2 | 6/4 | | 0.628 |

W/M/P, well-/moderately-/poorly-differentiated.

^a^Continuous values are means ± standard deviation; values in parentheses are percentages.

^b^All parameters were compared using Pearson’s χ^2^ and unpaired *t* tests.

^c^Pathological cancer staging according to the American joint committee on cancer (8th ed., 2017).

^d^Right, cecum – transverse colon; left, descending colon – sigmoid colon.
